# Prevention of suicides associated with global warming: perspectives from early career psychiatrists

**DOI:** 10.3389/fpsyt.2023.1251630

**Published:** 2023-11-14

**Authors:** Sheikh Shoib, Syed Sameer Hussaini, Aishatu Yusha’u Armiya’u, Fahimeh Saeed, Dorottya Őri, Thiago Henrique Roza, Ahmet Gürcan, Aditi Agrawal, Mireia Solerdelcoll, Don Eliseo Lucero-Prisno III, Mahsa Nahidi, Sarya Swed, Saeed Ahmed, Miyuru Chandradasa

**Affiliations:** ^1^Department of Health Services, Srinagar, India; ^2^Sharda University, Greater Noida, India; ^3^Psychosis Research Centre, University of Social Welfare and Rehabilitation Sciences, Tehran, Iran; ^4^Healing Mind and Wellness Initiative, Nawab Bzar, Srinagar, India; ^5^Ramaiah Medical College, Bengaluru, Karnataka, India; ^6^Department of Psychiatry, College of Medical Sciences, Abubakar Tafawa Balewa University, Bauchi, Nigeria; ^7^Institute of Behavioural Sciences, Semmelweis University, Budapest, Hungary; ^8^Department of Mental Health, Heim Pal National Paediatric Institute, Budapest, Hungary; ^9^Department of Psychiatry, Federal University of Rio Grande do Sul, Porto Alegre, Brazil; ^10^Department of Psychiatry, Başkent University Medical Faculty, Ankara, Türkiye; ^11^St Elizabeth Medical Centre, Boston University Affiliate, Boston, MA, United States; ^12^Department of Child and Adolescent Psychiatry, Institute of Psychology, Psychiatry and Neuroscience, King’s College London, London, United Kingdom; ^13^Department of Child and Adolescent Psychiatry and Psychology, Institute of Neuroscience, Hospital Clínic de Barcelona, Barcelona, Spain; ^14^Department of Global Health and Development, London School of Hygiene and Tropical Medicine, London, United Kingdom; ^15^Psychiatry and Behavioral Sciences Research Center, Mashhad University of Medical Sciences, Mashhad, Iran; ^16^Faculty of Medicine, Aleppo University, Aleppo, Syria; ^17^Rutland Regional Medical Centre, Rutland, VT, United States; ^18^Department of Psychiatry, University of Kelaniya, Ragama, Sri Lanka

**Keywords:** suicide, prevention, global warming, perspective, early career psychiatrists

## Abstract

Climate change poses significant challenges to global mental health, with potential consequences including increased rates of suicide and mental health disorders. Early Career Psychiatrists (ECPs) play a crucial role in addressing these challenges. The Climate Psychiatry Alliance, a group of psychiatrists dedicated to improving mental health amidst climate change, recognizes the importance of cultivating climate-aware ECPs. Training ECPs to become confident climate-aware clinicians enables them to effectively treat patients experiencing anxiety, depression, and PTSD in the context of climate-related distress. Together with other mental health professionals, ECPs can contribute to efforts by implementing strategies for monitoring and treating mental health problems arising from climate events. Additionally, they can raise awareness about the psychological consequences and risks of suicide associated with climate change. Collaboration among ECPs from various regions is essential in developing community-based approaches and reducing vulnerabilities. ECPs must prioritize supporting vulnerable populations by advocating for increased funding for mental health support and research in affected areas. Long-term solutions to address the mental health impacts of climate change and global warming should be pursued to mitigate future suicidality. Integrating climate considerations into local mental health programs and expanding psychological support services is crucial. By promoting emotional resilience and self-awareness, ECPs can contribute to building a more climate-resilient and mentally healthy society.

## Introduction

Global warming, characterized by rising temperatures, droughts, heat waves, disappearing rivers, devastating forest fires, melting glaciers, deserts, floods, and tornadoes, has become widely recognized as a significant environmental challenge (1). Over the past decade, our planet has experienced some of the highest recorded temperatures, accompanied by increased greenhouse gas emissions. In fact, six of the warmest years since the 1800s have occurred since 2014 (2). Furthermore, climate change-induced droughts have likely contributed to a surge in prolonged wildfires, leading to elevated levels of dust and smoke that pollute the air (3). These environmental transformations have a direct impact on the mental health of populations. Air pollution resulting from particulate matter has been linked to impaired cognition and memory, accelerated dementia, brain shrinkage, higher stroke rates, and other neurodegenerative processes in adults (4). Consequently, the increased frequency of natural disasters attributed to global warming imposes significant distress, affecting various mental health outcomes among affected populations (5).

### Global warming and mental health

Global warming leads to increases in temperature, sunlight exposure, air pollution, and changes in seasonal patterns, all of which could have implications for mental health. The rise in global temperatures is anticipated to result in an increase in the frequency of heat exposure.

A link between heat and heatwaves and mental health morbidity, such as admission to a mental hospital, emergency calls for mental health disorders, and psychiatric visits to the emergency room has been established in past research (6). This concurs with findings from studies conducted in Sweden (7) and Portugal (8). According to a study from Australia, along with other ailments such as cardiovascular and renal disease, heat waves are linked to higher rates of admissions for mental problems (9). Dementia, anxiety-related disorders, and mood disorders have all been linked to such heat waves (10).

Excessive heat exposure can cause both physical and mental fatigue (10). According to a study from Thailand, workplace heat stress is linked to greater psychological suffering in the workers (11). Similar findings have been described by other studies, which link higher workplace temperatures with increased psychological distress (12). The discomfort experienced during hot weather can lead to increased hostility, aggressive thoughts, and potentially aggressive behaviors. A study conducted in Spain revealed a correlation between heat waves and a rise in intimate partner violence (13). Indeed, high temperatures can induce physical and psychological exhaustion, and there is a significant association between increasing suicide rates and rising temperatures, especially during the peak of early summer (1).

Another potential point to consider is the association between rising temperatures and sleep difficulties. Sleep is fundamentally tied to body cooling (14). Cao et al. (15) found temperature to be the most important factor in sleep quality among the selected variables (15). Moreover, there is empirical evidence connecting higher temperatures with sleep problems and insufficient sleep, with this impact being more pronounced in people living in lower income countries, the elderly and women (16). Similarly, In addition, rising temperatures may lead to delayed onset of sleep and sleep loss (16). Importantly, poor sleep has been associated with several medical and psychiatric adverse outcomes, such as reduced productivity and cognitive performance, cardiovascular problems, depression and irritability, suicidality, among others (16).

Droughts are projected to worsen as a result of global climate change in the future (10). Increased floods are likely to result from changes in precipitation patterns in some regions, while extended droughts are predicted for others. Past research has found a link between farmer suicides and droughts (17). This trend has been observed in both industrialized nations such as Australia and developing nations such as India (10). There is a correlation between crop failures brought on by unforeseen droughts and farmer suicide attempts. The farmer may not be able to support the family’s expenses during dry conditions and may fall into debt. Moreover, droughts are often accompanied by warmer weather, which has been linked to increased suicide attempts. Lastly, protracted droughts lead to relocation and/or changes in line of work. The resulting acculturation stress is a risk factor for suicide.

Urban areas are also vulnerable to droughts. For instance, dry seasons associated with global warming and climate change may lead to shortage in water supplies (18). In addition, droughts in urban areas and cities are associated with reduced productivity, economic problems, and the spread of infectious diseases (19). These adverse consequences associated with droughts in urban areas are more likely to affect those economically disadvantaged (20). In this sense, these factors are likely to exert a significant burden in mental health outcomes as well.

A number of systematic reviews have addressed the impact of climate change on mental health. In a review of threats arising from climate change, Palinkas and Wong (21) found that anxiety, PTSD, and depression were the most common psychological impacts of climate change. They also found children to be especially susceptible (21). In a review of 163 studies, Cianconi et al. (1) found that different climatic events lead to different impacts among the affected populations, with temporally disparate timelines. For instance, heatwaves can have immediate direct effects on mental health, while sea level rise has indirect effects in the long term (1). A review of the mental health burden of climate change in Africa highlights the consequences of climate change in African countries, including hopelessness and helplessness, leading to stress and substance use. Children are especially vulnerable to climate change, as environmental changes can cause droughts, famines, and forced migrations, threatening children’s physical, emotional, and intellectual development through malnutrition, trauma, and diseases (22). A systematic review and meta-analysis of 53 studies focused on the mental health outcomes associated with heat waves found 2.2 and 0.9% increase in mental health-related mortality and morbidity for each 1°C increase in temperature, respectively (23).

It is also essential to highlight the potential differential impact that global warming and climate change may pose on indigenous communities. These communities are vulnerable, and already disadvantaged when it comes to poorer mental health outcomes; in addition, indigenous people are reliant on natural resources and on their land for survival, with this variable being a key factor for their mental health and wellbeing (24). All these factors may lead them to feel more strongly the impacts of climate change (25), the reason why special attention must be devoted to these populations.

### Climate change and the risk of suicide

#### Temperature

Considering the aforementioned factors, it is highly likely that the combination of these causes will contribute to an increase in suicidal behavior and suicide rates worldwide. Temperature and the suicide rates have been found to be related. With the recent rise in temperatures, it has been observed that suicides–especially violent suicides–are becoming more common (10). Higher temperatures were significantly positively associated with violent suicides (26) and suicide attempts (27), especially among males, according to two time-series studies that used national data on suicide and suicide attempts between 1984 and 1995. A subsequent time-series study using a comparable methodology and national suicide data for 1974–2003 found a reverse relationship between increasing anomalies in monthly average temperatures and a higher monthly suicide rates among males from May to August and to a lesser extent in November and December (28).

Deisenhammer et al. reviewed 27 studies on the relationship between attempted or completed suicide and meteorological factors. Most of the studies found some association between meteorological factors and suicides (29). In a study spanning some of the most populous counties in the United states, Dixon and Kalkstein (30) found an association between above-average temperatures and increased suicide risk, both in mid-latitude and sub-tropical cities (30). A study on suicides in São Paulo, Brazil, between 1996 and 2011 found that there was an immediate (<7 days) association between minimum temperatures and suicides by men. A study spanning 1751–2008 in Finland found that variations in temperature explain more than 60% of the total variance in suicides (31). Aguglia et al. (32) also report a significant association between violent suicides and apparent temperatures (29, 32). A 1°C increase in air temperature has been associated with a 1.1–2.3% increase in suicides (33–36). In a 2022 study, Giacomini et al. found an increase in suicides in warmer months due to global warming and report that climatic factors were a greater determinant of suicidal behavior compared with economic factors (37). Using an integrated health impact assessment model, Belova et al. (38) found that warming of 1–6°C could result in an annual increase of 283–1,660 deaths in coterminous United States (38). Similarly, Odabaşı found average daily minimum temperature to be a determinant of suicides across United States counties (39). A 2022 study from Belgium also found a significant association between higher temperatures and suicides compared to the median temperature, but not between colder temperatures and suicide (40). A meta-analysis by Kim et al. (41) encompassing over 1.3 million suicides in 12 countries across three continents found that warmer temperatures were associated with the overall suicide risk (41). A 2019 systematic review and meta-analysis found that a 1°C rise in temperature was correlated with a 1% rise in the occurrence of suicide. The correlation held whether the study had used mean temperature or the maximum (42).

A number of mechanisms have been proposed to explain the relationship between temperature and suicide rates. Postolache et al. (43) point out that dopamine, norepinephrine, and serotonin are involved in the regulation of mood and body temperature (43); responsivity and density of serotonin receptors are particularly affected by temperature (44–49). Helama et al. (31) propose a potential link between psychiatric conditions and altered transmission in the CNS projecting from brown adipose tissue through the hypothalamus to the periaqueductal gray areas. Given the role of cerebral structures in cognitive process, their reduced function could lead to more impulsive behaviors such as suicide (50). Another possible mechanism of action for temperature is the impact of temperature on the chance of survival following a suicide attempt (28).

#### Seasonality

Seasonal patterns are expected to shift due to climate change (51, 52). At the same time, several studies have found seasonal patterns in suicidal behavior. For instance, suicide rates peaked in late spring to early summer in nine counties across the US regardless of local climate (30). A similar pattern is reported by Jee et al. (53) for suicides during 1992–2010 in South Korea (53). Aguglia et al. (54) report that suicide attempts were more frequent during spring among patients who were involuntarily admitted to an Italian hospital (54). A 2018 systematic review including 45 studies from 26 countries and over 2 million suicides found clear seasonal and monthly patterns in suicides, mostly concentrated in the northern hemisphere’s spring and summer. Of the 27 studies that investigated suicides on a monthly basis, 22 found peaks in spring and early summer; furthermore, 13 studies investigating seasonal patterns found similar results (55). As global warming exacerbates across the world, the observed seasonal association between warmer temperatures and suicides might exacerbate due to the warmer temperatures throughout the year, in particular during the warmer months, given the established link between temperature and suicide.

Psychiatric and affective disorders tend to peak during spring and summer (56, 57). One hypothesis for this seasonal association implicates underlying seasonal biological variations which influence impulsivity and serotonin pathways (58). It has been reported that serotonin concentration is sensitive to climatic factors, which could influence the seasonal patterns in suicides (59). Along the same lines, it has been suggested that seasonal variations in sunlight affect serotonin levels and interactions (60). Another potential pathway is the negative effect of longer periods of light in spring and summer on circadian rhythm and sleep–wake cycle, leading to poor impulse control and increased suicidal behaviors (61, 62). Lower melatonin levels have also been suggested as a potential explanation for the seasonal peak in suicides (63). Spring and summer are also associated with warmer temperatures, which have been shown to be associated with increased suicide risk (31, 42, 64, 65).

Among the available studies, some have taken note of the differential influence of meteorological factors on violent vs. non-violent suicide attempts. For instance, Aguglia et al. (61) found that violent suicides were associated with summer and more intense solar radiation (61). Another study found a stronger correlation between apparent temperature, male gender, and violent suicides (32). A review study found the seasonal peak in suicides to be stronger among men and suicides committed using violent methods (66). A study on suicides between 1990 and 1999 in Victoria, Australia also found the seasonal pattern to be more pronounced for violent suicides (67). Preti and Miotto (26) observed a seasonal pattern only in violent suicides (26). Sergei et al. (68) also report that sunshine had a stronger effect on the risk of violent suicides, both in the total sample and among men (68). Two studies in Belgium also observed a significant positive association between violent suicides, temperature, and sunlight duration (69, 70).

A recent study examining the impact of climate change on fatal self-harm rates suggests that relative humidity has a stronger association with suicide compared to heatwaves (71). A systematic review also points to humidity and precipitation as potential correlates of suicide risk (29). These findings highlight the importance of considering various climatic factors when investigating the relationship between climate change and suicide rates. Moreover, cultural and sociological justifications have also been proposed for the association between meteorological factors and suicide. For instance, the impact of temperature has alternatively been explained as a consequence of increased alcohol consumption on warmer days, and its subsequent effects on the CNS (72). Additionally, the seasonal pattern has been attributed to events such as the start of the school year or the stress from the harvest season, as well as increased access to harmful chemicals (30).

The consequences of climate change can be intertwined with poor economic prospects in affected communities. For instance, a study conducted in rural India reported a 20% increase in suicide fatalities among adults during growing seasons with extremely wet or dry conditions, which negatively affected farmers who rely on agriculture for their livelihoods (73). Droughts have made farmers worldwide more susceptible to mental health issues due to concerns about their future (74), particularly among the elderly population (75).

## Discussion

### Interventions and coping strategies

Given the potential increase in suicides and suicidal behavior associated with global warming and climate change, it is crucial to implement urgent interventions and efforts to prevent future suicide attempts. Promoting positive mental health is paramount in addressing the psychological distress caused by climate change. Individuals can benefit from strengthening their coping mechanisms and building resilience to mitigate heightened stress levels. Practices such as yoga, exercise, and, importantly, raising awareness and providing access to programs that help individuals manage stress are all crucial.

Local health departments can play a vital role in supporting vulnerable communities by implementing actionable measures to improve access to mental health services, an intervention that has been shown to reduce suicidal behaviors (76). Additionally, local government organizations can address climate change-related suicidality by expanding financial support and safety nets for specific populations, such as farmers and other workers in the agriculture sector, who face economic hardships due to climate-related disasters (3). Implementing aggressive efforts to reduce greenhouse gas emissions will also contribute to mitigating the adverse effects of climate change on mental health and lowering the risk of catastrophic climate events (1). Other interventions include early response initiatives, such as allocating public funds for mental health support in the aftermath of devastating catastrophes.

Aid organizations such as the International Federation of Red Cross and Red Crescent Societies provide valuable training on recognizing and responding to the mental and physical distress caused by weather-related disasters (77). However, it is unfortunate that mental health services are often underfunded and overwhelmed in many parts of the world. As government funds are primarily allocated to front-line disaster relief, communities often bear the burden of responding to these challenges. Therefore, strengthening social connections within and outside communities becomes crucial in enhancing resilience to climatic disasters (77).

Climate change can trigger several mental health consequences such as PTSD, depression, anxiety, phobias, and sleep disorders, especially in more vulnerable groups such as children, older adults, and individuals with prior mental health problems (78–80). Therefore, psychosocial and community resilience may play a significant role in reducing the suffering associated with climate change and natural disasters, considering the importance that this concept presented during the COVID-19 pandemic (81, 82) In this sense, it is vital to prioritize the immediate restoration of social cohesion within communities and families to mitigate suffering and foster successful recovery. By fostering social support networks and promoting community engagement, individuals and communities can better cope with the adverse effects of climate change and build greater resilience. Efforts should be made to ensure that mental health support is integrated into disaster response and recovery plans to address the psychosocial needs of those affected. In addition, cultural aspects should be considered when promoting psychosocial resilience.

Community resilience serves as a preventative approach by preparing people for future disasters and supporting them in dealing with the present challenges. Strengthening the local community can be achieved through daily interactions with neighbors, participation in community organizations, and building relationships with local officials (83). Social support is strongly linked with resilience in the face of natural disasters (84–86). Social connectedness was also found to buffer against the effects of floods and droughts (87). Masten (88) found a number of factors contributing to individual and family resilience, including problem solving, flexibility, hope, optimism, positive self-perception, and emotional security (88). Among predictors of community resilience, research has found social capital to be an important factor as it provides access to resources and support in the face of climate change (84). Moreover, community cohesion promotes resilience against disasters (89).

Fostering psychological and community resilience requires a comprehensive set of solutions and recognition of the challenges posed by climate change and its wide-ranging implications. Coping mechanisms need to be developed to address the emotions and thoughts that arise, enabling individuals to acknowledge and confront the challenges and impacts of climate change rather than denying or ignoring them. Psychological, behavioral, social, and cognitive engagement is necessary, involving changes in behavior and lifestyle to protect oneself from the threats. Capacity building, training, and re-training individuals in psychological first aid practices are also crucial aspects of addressing mental health in the context of climate change (90).

In today’s digital era, the availability of mobile applications that deliver mental health content to users can be a valuable intervention strategy. Accessible alternatives like these can benefit many individuals, but it is important to ensure that these programs are tailored to incorporate different languages, cultural perspectives, and geographical features to better serve diverse users (91). By leveraging technology and considering the diversity of needs, mental health support can be made more accessible and effective in addressing the challenges posed by climate change.

### Early career psychiatrists and the climate psychiatry alliance

Early Career Psychiatrists (ECPs) should be aware of and actively address the potential impact of global warming on mental health. The Climate Psychiatry Alliance is a group of psychiatrists dedicated to enhancing mental well-being amidst climate change [*Climate Psychiatry* (92)]. The Climate Psychiatry Alliance acknowledges the importance of nurturing psychiatrists who are well-informed about climate-related issues and strives to involve ECPs in its endeavors. Training ECPs to enhance their confidence as climate-aware clinicians will enable them to effectively assist patients grappling with anxiety, depression, and PTSD in the current landscape (93). Through collaboration, mental health professionals can contribute to ongoing efforts by implementing diverse strategies and practices to monitor and address mental health issues that arise following acute climate events. Additionally, they can help raise awareness about the psychological consequences and risks of suicides associated with climate change (21). Together, these efforts can foster progress in safeguarding mental well-being amidst the challenges of a changing climate.

ECPs should recognize the importance of assisting vulnerable populations during the global battle against climate change. It is crucial to allocate increased funding for mental health support in affected areas and conduct research on this topic. By implementing sustainable solutions, we can prevent future instances of suicidality resulting from climate change and global warming. ECPs should prioritize the promotion of emotional resilience and self-awareness among their patients, while psychiatrists must integrate climate considerations into local mental health programs and expand psychological support services. Collaboration among ECPs from diverse regions is essential to strengthen global commitments and develop community-based approaches that reduce vulnerabilities. Together, these efforts can contribute to addressing the mental health challenges arising from climate change and building a more resilient future.

## Data availability statement

The original contributions presented in the study are included in the article/supplementary material, further inquiries can be directed to the corresponding author.

## Author contributions

ShS: conceptualization and writing the first draft. SH, AYA, FS, DŐ, TR, AG, AA, MS, DL-P, MN, SaS, SA, and MC: writing, revising, and editing the manuscript. All authors contributed to the article and approved the submitted version.
